# Association between vitamin D deficiency and type 2 diabetes in an Indigenous Brazilian population

**DOI:** 10.20945/2359-4292-2026-0084

**Published:** 2026-07-27

**Authors:** Hully Cantão dos Santos, Rafael de Oliveira Alvim, Gabriela Callo Quinte, Carolina Perim de Faria, Adriana Santos da Silva, José Geraldo Mill

**Affiliations:** 1 Universidade Federal do Espírito Santo, Programa de Pós-graduação em Saúde Coletiva, Vitória, ES, Brasil; 2 Universidade Federal do Amazonas, Departamento de Ciências Fisiológicas, Manaus, AM, Brasil; 3 Universidade Federal do Espírito Santo, Departamento de Nutrição, Vitória, ES, Brasil; 4 Universidade Federal do Espírito Santo, Departamento de Ciências Fisiológicas, Vitória, ES, Brasil

**Keywords:** Diabetes mellitus, type 2, vitamin D deficiency, Indigenous peoples

## Abstract

**Objective:**

This study investigated the association between vitamin D levels and type 2 diabetes mellitus (T2DM) among Indigenous adults of the Tupiniquim and Guarani ethnicities residing on a reserve on the southeastern Brazilian coast.

**Subjects and methods:**

This cross-sectional study, conducted between 2020 and 2022, included 946 Indigenous individuals (aged ≥20 years, both sexes) from the Tupiniquim and Guarani ethnic groups. T2DM was defined by a self-reported diabetes diagnosis, the use of glucose-lowering medications, or, in the absence of such reports, a fasting plasma glucose level ≥126 mg/dL, a 2-hour plasma glucose level ≥200 mg/dL following an oral glucose tolerance test, or a glycated hemoglobin level ≥6.5%. Hypovitaminosis D was defined as a serum 25-hydroxyvitamin D [25(OH)D] level <30.0 ng/mL. Associations were estimated using multiple logistic regression models.

**Results:**

The mean age of the participants was 41.3 ± 14.9 years; 56.8% were women, and 90% belonged to the Tupiniquim ethnic group. After adjusting for confounders, serum 25(OH)D levels were inversely associated with T2DM. Each 1-ng/mL increase in 25(OH)D was associated with lower odds of T2DM (OR = 0.96; 95% CI: 0.94-0.98). Individuals with hypovitaminosis D had higher odds of T2DM than those with adequate vitamin D levels (OR = 1.77; 95% CI: 1.20-2.62).

**Conclusion:**

Vitamin D deficiency was independently associated with the presence of T2DM. The high prevalence of hypovitaminosis D underscores the importance of screening strategies and considering supplementation protocols, even among Indigenous populations with high solar exposure.

## INTRODUCTION

Type 2 diabetes mellitus (T2DM) is a chronic, multifactorial disease characterized by persistent hyperglycemia resulting from insulin resistance in peripheral tissues (^1^). Over the past decades, the global prevalence of T2DM has more than doubled, and it is estimated that by 2030, over 600 million adults aged 20-79 years will be living with the disease worldwide (^2^,^3^). In Brazil, the prevalence of T2DM has been estimated at 10.6% (^4^). Among the Brazilian Indigenous population, the prevalence of T2DM is remarkably high. A review of studies published between 2011 and 2022 reported that the prevalence of T2DM ranged from 3.0% to 24.9% (^5^). Consistent with these findings, the prevalence of this disease among Indigenous people living in the Aracruz reserve in Espírito Santo State (southeastern Brazil), increased from 4.3% in 2003 to 13.7% in 2020 (^6^). The main factors associated with the high incidence of T2DM include older age (^7^), lifestyle behaviors (^8^), family history of the disease (^9^), obesity (^10^), hypertension (^11^), dyslipidemia (^12^), low educational attainment (^13^), unfavorable socioeconomic conditions (^8^,^13^) and low circulating levels of vitamin D (^14^,^15^).

Vitamin D is a fat-soluble prohormone essential for regulating calcium metabolism; it plays multiple roles across various tissues and organs, influencing the expression of genes related to the development of metabolic, hormonal, and immunological diseases (^16^). Only 10-20% of the daily vitamin D requirement is obtained through diet, while 80-90% depends on endogenous synthesis, which requires skin exposure to ultraviolet sunlight (^17^). In recent years, hypovitaminosis D has emerged as a relevant risk factor for the development of T2DM. Studies have consistently demonstrated an inverse association between serum levels of 25-hydroxyvitamin D [25(OH)D] (a biomarker of vitamin D status) and the risk of developing T2DM (^14^,^15^). Research conducted among Australian and Brazilian Indigenous populations has shown that individuals with lower 25(OH)D concentrations have a higher likelihood of developing T2DM (^18^,^19^). However, studies investigating this association, specifically among Indigenous populations living in tropical regions of Brazil, remain scarce.

Considering the increasing prevalence of T2DM among Indigenous peoples in Brazil, the relevance of vitamin D as a potentially modifiable risk factor, and the scarcity of studies investigating this association in this population, we aimed to analyze the association between vitamin D levels and T2DM in Indigenous adults of the Tupiniquim and Guarani ethnicities living in a reserve on the southeastern Brazilian coast.

## SUBJECTS AND METHODS

### Study design and population

This cross-sectional study used data from the survey “Evaluation of the Prevalence and Gravity of Chronic Diseases in the Indigenous Population of Espírito Santo”, conducted at the University Hospital (HUCAM) of the Federal University of Espírito Santo. The officially demarcated Indigenous lands (Comboios, Caieiras Velha II, and Tupiniquim), located in the municipality of Aracruz on the northern coast of Espírito Santo state, are home to Indigenous people distributed across 12 villages of the Tupiniquim and Guarani ethnicities. The Tupiniquim, who belong to the Tupi-Guarani linguistic family, predominantly use Portuguese in their daily lives as a result of historical colonization and interethnic contact, alongside contemporary initiatives for cultural revitalization. The Guarani, mostly from the Mbya subgroup, preserve the Guarani language as their native tongue; it is widely used in social, educational, and spiritual practices, allowing them to maintain strong community organization and a distinct cultural identity. Both groups, however, maintain close contact with the general population living outside the reserve, with unrestricted access to commercial establishments such as shops and supermarkets.

Data collection was carried out between September 2020 and July 2022. All adults residing in the Indigenous reserve (approximately 2,500 individuals) in February 2020 were invited to the HUCAM outpatient clinic to undergo clinical and laboratory testing for cardiovascular risk factors. A subsample of 1,084 individuals attended the hospital clinic. For the present analysis, we excluded 110 participants who self-identified as non-Indigenous, 13 from ethnicities other than Tupiniquim and Guarani, 5 due to current vitamin D supplementation, 4 due to missing data on vitamin D levels, and 6 diagnosed with type 1 diabetes mellitus. Thus, the final sample consisted of 946 individuals. A detailed description of the research design and the main characteristics of the study population has been previously published (^6^).

This study was approved by the Research Ethics Committee (CAAE no. 22563019.6.0000.5060, approval no. 3.655.623) of the Health Sciences Center at the Federal University of Espirito Santo and the Brazilian National Research Ethics Commission (CAAE no. 22563019.6.0000.5060, approval no. 3.828.655). All participants signed the informed consent form before data collection.

### Questionnaire application

Sociodemographic and lifestyle variables were collected during interviews using a standardized questionnaire. The variables included: sex (male or female), age (in years), and ethnicity (Tupiniquim or Guarani). The level of education (low, medium, or high) was categorized based on years of schooling (≤8, 9-11, and ≥12 years, respectively). Smoking status (never, former, or current) and alcohol consumption (consumer or non-consumer) were also determined during the interview. Seasonality was classified based on the month of data collection: summer (December, January, and February); autumn (March, April, and May); winter (June, July, and August); and spring (September, October, and November).

### Biochemical measurements

Fasting blood samples were collected via venipuncture after a 12- to 14-hour fasting period to measure glucose, glycated hemoglobin (HbA1c), 25(OH)D, total cholesterol, cholesterol fractions, and insulin. Participants without a previous diagnosis of diabetes mellitus underwent a standard oral glucose tolerance test, which consisted of ingesting a flavored solution containing 75 g of glucose. Two hours after ingestion, a second blood sample was collected to measure plasma glucose levels. The samples were centrifuged and aliquoted at the collection site and subsequently transported to the clinical analysis laboratory at HUCAM. Fasting glucose, triglycerides, and HDL-c were measured using an enzymatic colorimetric method; HbA1c was measured using liquid chromatography with boronate affinity; and LDL-c was calculated using the Friedewald formula. Plasma concentrations of 25(OH)D were determined using a commercial Architect Abbott kit based on a chemiluminescent immunoassay method.

The presence of diabetes mellitus was defined according to the following criteria: self-reported diagnosis of diabetes, self-reported use of glucose-lowering medications, or, in the absence of such reports, fasting plasma glucose ≥126 mg/dL, plasma glucose 2 hours after the oral glucose tolerance test ≥200 mg/dL, or HbA1c ≥6.5% (^1^). Dyslipidemia was defined as self-reported use of lipid-lowering medications (statins, fibrates, or ezetimibe) or the presence of abnormal lipid levels, specifically LDL-c ≥160 mg/dL, triglycerides ≥150 mg/dL, or HDL-c <40 mg/dL for men and <50 mg/dL for women, according to the Brazilian Guidelines for Dyslipidemia and Atherosclerosis Prevention (^20^).

In this study, vitamin D was analyzed both as a continuous and a categorical variable. For the categorical classification, the following cut-off points were adopted: deficiency for concentrations <20 ng/mL, insufficiency between 21-29 ng/mL, and sufficiency for values ≥30 ng/mL (^21^). For analytical purposes, vitamin D status was dichotomized into adequate (≥30 ng/mL) and hypovitaminosis D (<30 ng/mL).

### Blood pressure measurements

Blood pressure was measured on the left arm after a rest period of at least 5 minutes in a quiet, temperature-controlled environment (20-24 °C) using a validated oscillometric device (Omron HEM 705CPINT). Three measurements were taken at approximately one-minute intervals, and the average of the last two readings was recorded (^22^). Hypertension was defined as blood pressure ≥140/90 mmHg or self-reported continuous use of antihypertensive medication, including diuretics (^23^).

### Anthropometric evaluations

Body weight was measured while participants were fasting, barefoot, without accessories, after bladder emptying, and wearing a standard uniform (400-600 g) using an electronic scale (Toledo; https://www.toledobrasil.com/) with an accuracy of 0.1 kg. Height was measured using a fixed wall stadiometer (Seca; https://www.seca.com/pt_mz.html) with an accuracy of 0.1 cm. These measurements were used to calculate body mass index (BMI), defined as weight (kg) divided by height squared (m^2^) (^24^).

### Statistical analyses

Continuous variables were described using the mean and standard deviation or the median and interquartile range, as appropriate, while categorical variables were presented as absolute and relative frequencies. The normality of continuous variables was assessed using the Kolmogorov-Smirnov test. Comparisons between groups for continuous variables were performed using the independent samples Student’s *t*-test or, when normality was not met, the Mann-Whitney U test. Differences in categorical variables were assessed using the chi-square test. Multiple logistic regression was conducted to identify independent associations between 25(OH)D levels (ng/mL) or vitamin D status and the presence of diabetes. Covariates were selected based on biological plausibility and statistical significance observed in the bivariate analyses between groups with and without hypovitaminosis D, as well as between participants with and without T2DM. Therefore, the variables included in the model were: sex, age, level of educational, smoking, alcohol consumption, BMI, dyslipidemia, hypertension, and season of the year. Participants without diabetes were the reference group. All statistical analyses were performed using Stata software v. 18. A *p*-value <0.05 was considered statistically significant.

## RESULTS

The general characteristics of the sample, stratified by sex, are summarized in **Table 1**. The frequencies of smoking and alcohol consumption were higher among men. Moreover, men were older and exhibited higher levels of 25(OH)D, fasting blood glucose, systolic blood pressure, and diastolic blood pressure. In contrast, the frequencies of diabetes, dyslipidemia, and obesity were higher among women, who also presented higher HDL cholesterol levels and BMI.

**Table 1:** Sociodemographic, clinical, and lifestyle characteristics of the sample

Variables	Total (n = 946)	Men (n = 409)	Women (n = 537)	*p*-value
Age, years	41.3 ± 14.9	43.1 ± 15.1	39.9 ± 14.5	<0.01
Ethnicity, n (%)				
Tupiniquim	850 (90.0)	376 (92.2)	474 (88.4)	0.06
Guarani	94 (10.0)	32 (7.8)	62 (11.6)	
Level of education, n (%)				
Low	302 (31.9)	138 (33.7)	164 (30.6)	0.18
Medium	546 (57.8)	237 (58.0)	309 (57.6)	
High	97 (10.2)	34 (8.3)	63 (11.8)	
25(OH)D, ng/mL	32.8 ± 9.9	33.6 ± 10.6	32.1 ± 9.3	0.02
Hypovitaminosis D, n (%)	413 (43.7)	167 (40.8)	246 (45.8)	0.13
Fasting blood glucose, mg/dL	98 (91-108)	100 (94-110)	97 (89-107)	<0.01
2-Hour glucose, mg/dL	126 (103.5-157)	116 (99-145.5)	134 (108-163)	<0.01
HbA1c, %	6.0 ± 1.6	5.9 ± 1.4	6.1 ± 1.7	0.15
BMI, kg/m^2^	29.3 ± 5.8	27.7 ± 4.7	30.5 ± 6.3	<0.01
SBP, mmHg	126.4 ± 17.7	130.5 ± 16.1	123.3 ± 18.2	<0.01
DBP, mmHg	75.5 ± 9.7	76.6 ± 9.7	74.6 ± 9.5	<0.01
HDL-cholesterol, mg/dL	45.4 ± 11.3	44.5 ± 11.9	46.1 ± 10.7	0.03
LDL-cholesterol, mg/dL	116.1 ± 36.3	118.3 ± 37.1	114.5 ± 35.7	0.11
Total cholesterol, mg/dL	190.9 ± 42.7	192.7 ± 43.2	189.6 ± 42.3	0.26
Triglycerides, mg/dL	124 (85-185)	128 (84-195)	122 (85-170)	0.19
Dyslipidemia, n (%)	671 (71.5)	258 (63.9)	413 (77.3)	<0.01
Hypertension, n (%)	326 (34.5)	146 (35.7)	180 (33.5)	0.48
T2DM, n (%)	180 (19.0)	64 (15.6)	116 (21.6)	0.02
Obesity, n (%)	369 (39.0)	109 (26.6)	260 (48.4)	<0.01
Smoking, n (%)	145 (15.3)	85 (20.8)	60 (11.2)	<0.01
Alcohol use, n (%)	390 (41.4)	191 (46.8)	199 (37.3)	<0.01

Data are expressed as mean ± standard deviation or median (interquartile range) or n (%). HbA1c: glycated Hb; BMI: body mass index; SBP: systolic blood pressure; DBP: diastolic blood pressure; HDL-c: high-density lipoprotein; LDL-c: low-density lipoprotein; T2DM: type 2 diabetes mellitus.

Table 2 presents an analysis of sociodemographic, clinical, and lifestyle characteristics according to vitamin D status. Participants with adequate 25(OH)D levels demonstrated a lower frequency of diabetes and obesity, alongside a higher proportion of smokers and individuals of Guarani ethnicity. Furthermore, this group presented significantly lower fasting blood glucose and HbA1c levels compared to those with hypovitaminosis D. The season of data collection was not significantly associated with vitamin D status.

**Table 2:** Sociodemographic, clinical, and lifestyle characteristics according vitamin D status of the sample.

Variables	Adequate vitamin D (n = 533)	Hypovitaminosis D (n = 413)	*p*-value
Age, years	39.9 ± 14.8	40.0 ± 14.2	0.90
Ethnicity, n (%)			
Tupiniquim	467 (87.8)	383 (93.0)	0.01
Guarani	65 (12.2)	29 (7.0)	
Level of education, n (%)			
Low	177 (33.2)	125 (30.3)	0.62
Medium	304 (57.0)	242 (58.7)	
High	52 (9.8)	45 (10.9)	
Fasting blood glucose (mg/dL)	97 (90-106)	98 (92-113)	0.01
2-Hour glucose, mg/dL	121 (100-154)	130 (108-160)	0.01
HbA1c, %	5.9 ± 1.4	6.1 ± 1.8	0.04
BMI, kg/m^2^	28.5 ± 5.4	30.3 ± 6.2	<0.01
SBP, mmHg	126.6 ± 18.1	126.0 ± 17.1	0.58
DBP, mmHg	75.5 ± 9.8	75.5 ± 9.4	0.94
HDL-cholesterol, mg/dL	45.1 ± 11.5	45.8 ± 11.1	0.37
LDL-cholesterol, mg/dL	117.5 ± 37.5	114.3 ± 34.7	0.19
Total cholesterol, mg/dL	190.9 ± 43.8	190.1 ± 41.3	0.60
Triglycerides, mg/dL	123 (83-184)	127 (85-185)	0.38
Dyslipidemia, n (%)	383 (72.7)	288 (70.1)	0.38
Hypertension, n (%)	195 (36.6)	131 (31.7)	0.12
T2DM, n (%)	87 (16.3)	93 (22.5)	0.02
Obesity, n (%)	188 (35.3)	181 (43.8)	0.01
Smoking, n (%)	95 (17.8)	50 (12.1)	0.04
Alcohol use, n (%)	233 (43.9)	157 (38.3)	0.08
Seasons n (%)			
Spring/summer	280 (52.5)	211 (51.1)	0.66
Autumn/winter	253 (47.5)	202 (49.9)	

Data are expressed as mean ± standard deviation or median (interquartile range) or n (%). HbA1c: Glycated Hb; BMI: body mass index; SBP: systolic blood pressure; DBP: diastolic blood pressure; HDL-c: high-density lipoprotein; LDL-c: low-density lipoprotein; T2DM: type 2 diabetes mellitus.

Table 3 presents a detailed analysis of sociodemographic, clinical, and lifestyle characteristics according to diabetes status. As expected, participants with diabetes were older, had lower levels of education, and presented a less favorable lipid and hemodynamic profile. Additionally, the diabetic group showed lower 25(OH)D levels and higher frequencies of obesity and hypovitaminosis D. Conversely, the frequencies of alcohol consumption and smoking were lower among individuals with diabetes.

**Table 3:** Sociodemographic, clinical, and lifestyle characteristics according to diabetes status of the sample

Variables	Without diabetes (n = 766)	With diabetes (n = 180)	*p*-value
Age, years	38.6 ± 13.9	52.9 ± 13.2	<0.01
Ethnicity, n (%)			
Tupiniquim	682 (89.3)	168 (93.3)	0.10
Guarani	82 (10.7)	12 (6.7)	
Level of education, n (%)			
Low	214 (28.0)	88 (48.9)	<0.01
Medium	464 (60.6)	82 (45.5)	
High	87 (11.4)	10 (5.6)	
25(OH)D, ng/dL	33.3 ± 10.0	30.6 ± 8.9	<0.01
Hypovitaminosis D, n (%)	320 (41.8)	93 (51.7)	0.02
BMI, kg/m^2^	28.9 ± 5.8	31.0 ± 5.6	<0.01
SBP, mmHg	123.8 ± 15.9	137.3 ± 20.4	<0.01
DBP, mmHg	74.7 ± 9.3	79.0 ± 10.3	<0.01
HDL-cholesterol, mg/dL	45.9 ± 11.4	43.3 ± 10.6	0.01
LDL-cholesterol, mg/dL	115.0 ± 35.4	121.1 ± 39.8	0.05
Total cholesterol, mg/dL	187.9 ± 41.7	203.9 ± 44.6	<0.01
Triglycerides, mg/dL	116 (80-166)	176.5 (121.5-254.5)	<0.01
Dyslipidemia, n (%)	516 (68.1)	155 (86.1)	<0.01
Hypertension, n (%)	200 (26.1)	126 (70.0)	<0.01
Obesity, n (%)	273 (35.6)	96 (53.3)	<0.01
Smoking, n (%)	123 (16.1)	22 (12.2)	<0.01
Alcohol use, n (%)	331 (43.5)	59 (32.8)	0.01

Data are expressed as mean ± standard deviation or median (interquartile range) or n (%). HbA1c: Glycated Hb; BMI: body mass index; SBP: systolic blood pressure; DBP: diastolic blood pressure; HDL-c: high-density lipoprotein; LDL-c: low-density lipoprotein.

Univariate and multivariate logistic regression models were employed to examine the association between vitamin D and T2DM (**Table 4**). In the fully adjusted model, serum 25(OH)D concentration, treated as a continuous variable, was inversely associated with T2DM. Each 1 ng/mL increase in 25(OH)D corresponded to a 4% reduction in the odds of having diabetes. Furthermore, when vitamin D was categorized, individuals with hypovitaminosis D had 77% higher odds of having diabetes compared to those with adequate vitamin D levels.

**Table 4:** Association between vitamin D status/levels and diabetes mellitus of the sample

Vitamin D	Odds ratio	95% Confidence interval	p-value
Status			
Crude model			
Adequate vitamin D	1		
Hypovitaminosis D	1.49	1.07-2.06	0.02
Adjusted model			
Adequate vitamin D	1		
Hypovitaminosis D	1.77	1.20-2.62	<0.01
Level			
Crude model			
25(OH)D (ng/dL)	0.97	0.95-0.99	<0.01
Adjusted model			
25(OH)D (ng/dL)	0.96	0.94-0.98	<0.01

Adjusted model: sex, age, education, ethnicity, smoker, alcohol use, body mass index, dyslipidemia, hypertension, season.

## DISCUSSION

Our findings revealed that individuals with T2DM had significantly lower serum vitamin D concentrations than those without the disease. The high prevalence of T2DM and hypovitaminosis D among the participants is also noteworthy. Studies have shown a wide variation in the prevalence of T2DM among different Indigenous groups in Brazil, with rates ranging from 4.5% to 28.2% (^25^). In our study, the prevalence was 19%, falling within this range but remaining higher than the estimated 10.6% prevalence for the general Brazilian population (^4^). These findings underscore the importance of investigating potential contributing factors (e.g., hypovitaminosis D), which may play a role in T2DM development within this population.

The prevalence of hypovitaminosis D in the assessed Indigenous population was 43.8%, with 36.0% classified as insufficient and 7.7% as deficient (data not shown). In comparison with the available literature, our findings highlighted relevant particularities when contrasted with data from the Xavante Indigenous population. In this context, Abrahão and cols. (^18^) reported rates of 65% for insufficiency and approximately 28% for vitamin D deficiency among Xavante Indigenous individuals living in a large reservation in the state of Mato Grosso, in the Central-West region of Brazil. The substantial difference between these two studies may be attributed to factors such as geographical variations and differences in cultural and dietary habits. Moreover, our results were similar to those reported among Australian Indigenous populations in terms of insufficiency (42%) but lower regarding deficiency, which reached 31% in that population (^19^). These findings suggest that although vitamin D insufficiency is a common issue among different Indigenous groups, the magnitude of deficiency seems to vary substantially depending on environmental and sociocultural contexts. Furthermore, when comparing our results to those of the general population, the total prevalence of hypovitaminosis D was lower than that reported by Borba and cols. (^26^), who identified 15.0% insufficiency and 50.7% deficiency. This contrast suggests that certain Indigenous groups may benefit from greater sun exposure compared to the general population.

Within the study population, relevant differences in serum vitamin D levels were also observed between the Guarani and Tupiniquim ethnic groups. A higher proportion of Guarani individuals presented adequate vitamin D levels, which may be associated with the lower prevalence of overweight and obesity in this group compared to the Tupiniquim (62.8% [95% CI 60.1-65.5] *vs*. 78.0% [95% CI 75.3-80.7]; data not shown). In addition, the Guarani tend to maintain more traditional lifestyles, whereas the Tupiniquim exhibit a more urbanized lifestyle, which may negatively affect sun exposure and, consequently, serum 25(OH)D levels (^27^). These findings highlighted the importance of considering cultural and behavioral specificities when interpreting vitamin D nutritional status among Indigenous populations.

The association between hypovitaminosis D and higher serum HbA1c levels has been widely discussed in the literature, suggesting that vitamin D deficiency may contribute to poorer glycemic control (^28^). In this study, individuals with hypovitaminosis D showed significantly higher mean HbA1c levels than those with adequate vitamin D levels. Similar findings were reported by Bhat and cols. (^29^), who identified significantly higher 25(OH)D concentrations among individuals with good glycemic control (<7%) than those with poor glycemic control. Felício and cols. (^30^) also demonstrated that individuals with HbA1c ≥7% (i.e., poorer glycemic control) had significantly lower 25(OH)D concentrations than those with adequate glycemic control (<7%). These findings further support the hypothesis that vitamin D deficiency is associated with poorer glycemic control, thus predisposing individuals to diabetes development.

Regarding T2DM, we observed an inverse association between serum 25(OH)D levels and the presence of the disease, even after adjusting for potential confounding factors. These results are consistent with literature, which have identified vitamin D deficiency as a potentially modifiable factor in the pathogenesis of T2DM (^21^,^31^,^32^). This association has also been reported in studies conducted among Indigenous populations. In Brazil, Abrahão and cols. (^18^) found that higher 25(OH)D levels were significantly associated with a lower prevalence of diabetes mellitus and glucose intolerance among the Xavante Indigenous population. Specifically, each 1 ng/mL increase in serum 25(OH)D was associated with a 3% reduction in the likelihood of developing diabetes. Similarly, a study conducted with Australian Indigenous participants reported that lower 25(OH)D levels were associated with a higher prevalence of diabetes mellitus. Individuals with hypovitaminosis D had an odds ratio of 2.15 for diabetes, even after adjusting for cardiometabolic risk factors (^19^).

Proposed mechanisms for this association are based on a physiological perspective, wherein vitamin D plays a fundamental role in the regulation of glucose metabolism. It actively participates in pancreatic beta-cell function by acting as a chemical messenger through interaction with receptors that regulate calcium flux within beta cells, a process essential for insulin secretion (^33^). Additionally, vitamin D stimulates the expression of insulin receptors and activates the peroxisome proliferator-activated receptor delta, thereby promoting peripheral glucose uptake and improving insulin sensitivity. Moreover, vitamin D has been implicated in modulating inflammatory mechanisms and regulating the renin-angiotensin system, whose hyperactivity contributes to insulin resistance and endothelial dysfunction, both relevant factors in the pathophysiology of T2DM (^34^).

Our findings do not support the recommendation of universal vitamin D supplementation for all Indigenous individuals, nor do they imply a homogeneous classification of this population as high-risk for T2DM based solely on serum 25(OH)D levels. As highlighted by Fuentes-Barría and cols. (^35^), the American Diabetes Association does not currently recommend routine supplementation to prevent or treat T2DM, except in cases of laboratory-confirmed deficiency.

Nevertheless, our results indicated that low vitamin D levels were associated with a higher probability of T2DM in this specific context, reinforcing their clinical relevance as an indirect marker of metabolic health. Accordingly, rather than a mandate for mass supplementation, these data underscore the need for targeted clinical screening and public health strategies. Such efforts should focus on identifying the most vulnerable groups within Indigenous communities and promoting lifestyle-based interventions, including the preservation of traditional diets and adequate sun exposure, aligned with an evidence-based and individualized care approach.

A key strength of this study lies in the inclusion of a sample composed of Indigenous individuals living in villages with close interaction with urban environments, a reality that reflects the situation of many Indigenous populations in other regions of Brazil. Another noteworthy strength is the rigorous methodological approach employed during data collection, which contributes to the robustness of the results. Nevertheless, these findings must be interpreted cautiously given the potential limitations. First, the sample is not representative of the entire Indigenous population living in villages across Brazil; therefore, generalizing the findings should be approached with caution. In addition, the absence of relevant variables for understanding vitamin D levels (e.g., duration of sun exposure, sunscreen use, and type of clothing) may have limited the explanatory power of the results. Lastly, the inherent limitations of the cross-sectional study design prevent the establishment of causal relationships between the variables analyzed.

In conclusion, our findings reinforced the hypothesis that vitamin D deficiency was independently associated with the presence of T2DM. Furthermore, a high prevalence of hypovitaminosis D was observed in the study population, which reinforces the importance of screening strategies, as well as the definition of supplementation protocols even in Indigenous groups presumably exposed to high solar radiation.

## Data Availability

datasets related to this article will be avail-able upon request to the corresponding author.
